# 固相萃取-超高效液相色谱-串联质谱法测定生活饮用水中16种有机紫外吸收剂

**DOI:** 10.3724/SP.J.1123.2025.10015

**Published:** 2026-06-08

**Authors:** Zhuo WANG, Can ZHAO, Bixiong YE, Yongyan CHEN

**Affiliations:** 中国疾病预防控制中心环境与人群健康重点实验室，中国疾病预防控制中心环境与健康 相关产品安全所，北京 100050; China CDC Key Laboratory of Environment and Population Health，National Institute of Environmental Health，Chinese Center for Disease Control and Prevention，Beijing 100050，China

**Keywords:** 固相萃取, 超高效液相色谱-三重四极杆质谱, 有机紫外吸收剂, 饮用水, solid phase extraction （SPE）, ultra-high performance liquid chromatography-triple quadrupole mass spectrometry （UHPLC-MS/MS）, organic ultraviolet absorbers （OUVAs）, drinking water

## Abstract

有机紫外吸收剂（OUVAs）是一类近年来备受关注的新污染物。本研究针对水体中OUVAs检出率高且含量低的特点，建立了一种基于固相萃取-超高效液相色谱-串联质谱同时检测生活饮用水中9种紫外稳定剂与7种紫外过滤剂的分析方法。在500 mL水样中先加入25 mg抗坏血酸，然后加入盐酸将pH调至2~3，再加入1 mL 5 μg/L的混合内标工作液，用HLB固相萃取柱（200 mg/6 mL）对水样中的目标化合物进行富集浓缩，以Acquity Premier BEH C_18_（100 mm×2.1 mm，1.7 μm）色谱柱分离，甲醇-2 mmol/L乙酸铵水溶液为流动相进行梯度洗脱，采用电喷雾正离子模式电离、多反应监测模式检测，内标法定量。以饮用水作为基质，对所建立方法的精密度和准确度进行考察。结果表明，16种有机紫外吸收剂在一定范围内均呈现良好的线性关系，相关系数*r*均大于0.992。方法检出限（MDL，*S/N*=3）为0.03~5 ng/L，定量限（MQL，*S/N*=10）为0.1~15 ng/L。目标分析物在5、20、50 ng/L 3个加标水平下，回收率为75.0%~130.6%，相对标准偏差（RSD）为0.9%~12.9%（*n*=6）。用该方法对7份水源水样品和7份饮用水样品进行检测，实验结果表明，2-羟基-4-甲氧基二苯甲酮（UV-9）、奥克立林（OC）共2种有机紫外吸收剂被检出；水源水中UV-9的检出质量浓度为<MQL~13.4 ng/L，OC的检出质量浓度为5.0~32.23 ng/L。饮用水UV-9的检出质量浓度为0.4 ng/L，OC的检出质量浓度为<MQL~13.2 ng/L。该方法适用于生活饮用水中16种有机紫外吸收剂痕量分析测定，可用于我国生活饮用水中有机紫外吸收剂的环境监测分析。

## Abstract (en)

Organic ultraviolet absorbers （OUVAs） are a class of emerging contaminants that have garnered significant attention in recent years. In response to the characteristics of high detection frequency and low concentrations of OUVAs in water bodies， this study established an analytical method based on solid phase extraction （SPE） coupled with ultra-high performance liquid chromatography-tandem mass spectrometry （UHPLC-MS/MS） for the simultaneous determination of 16 OUVAs （nine UV stabilizers and seven UV filters） in drinking water. The 500 mL water sample was pretreated with 25 mg ascorbic acid， acidified to pH 2-3， and spiked with 1 mL of 5 μg/L mixed internal standard working solution. Target compounds were enriched and concentrated using an HLB solid phase extraction cartridge （200 mg/6 mL）. Separation was achieved on an Acquity Premier BEH C_18_ column （100 mm×2.1 mm， 1.7 μm） using a gradient elution with methanol and 2 mmol/L ammonium acetate aqueous solution as the mobile phases. Detection was performed using electrospray ionization in positive mode and multiple reaction monitoring （MRM）， with quantification carried out by the internal standard method. The precision and accuracy of the established method were evaluated using drinking water as the matrix. The results showed that all 16 OUVAs exhibited good linearity within their respective mass concentration ranges， with correlation coefficients （*r*） greater than 0.992. The method detection limits （MDL， *S/N=*3） were 0.03-5 ng/L， and the method quantification limits （MQL， *S/N=*10） were 0.1-15 ng/L. At spiked levels of 5， 20 and 50 ng/L， the recoveries of the target analytes ranged from 75.0% to 130.6%， with relative standard deviations （RSDs） ranging from 0.9% to 12.9% （*n*=6）. A total of seven source water samples and seven drinking water samples were analyzed using this method. The results showed that two OUVAs， 2-hydroxy-4-methoxybenzophenone （UV-9） and octocrylene （OC） were detected. In source water， the mass concentrations of UV-9 ranged from <MQL to 13.4 ng/L， while OC mass concentrations ranged from 5.0 to 32.23 ng/L. In drinking water， UV-9 was detected at 0.4 ng/L， and OC mass concentrations ranged from <MQL to 13.2 ng/L. This method is suitable for the trace analysis of 16 OUVAs in drinking water， demonstrating good accuracy and precision. It effectively improves the detection efficiency of OUVAs in water and can be used for the environmental monitoring and analysis of OUVAs in drinking water in China.

有机紫外吸收剂（organic ultraviolet absorbers，OUVAs）作为一类已知但近些年才被广泛认识其环境风险的化合物备受重视，已有研究表明OUVAs具有内分泌干扰、遗传毒性及生殖毒性等潜在危害^［1，2］^。例如，2-（2′-羟基-3′，5′-二叔丁基苯基）-苯并三唑（UV-320）和2-（2′-羟基-3′，5′-二叔戊基苯基）苯并三唑（UV-328）因具有强持久性、生物累积性和毒性，于2014年被欧洲化学品管理局列为高度关注物质，随后2-（2′-羟基-3′，5′-二叔丁基苯基）-5-氯代苯并三唑（UV-327）和2-（2′-羟基-3′-异丁基-5′-叔丁基苯基）苯并三唑（UV-350）也于2015年加入该名单^［3］^。UV-328作为常用苯并三唑类OUVAs，于2019年列入加拿大国内物质清单（DSL）^［4］^，并于2023年被提议纳入《斯德哥尔摩公约》附件A^［5］^。此外，欧洲化学品管理局（ECHA 2024）已将UV-320和2′-（2′-羟基-3′-叔丁基-5′-甲基苯基）-5-氯苯并三唑（UV-326）指定为高度关注物质^［3］^。OUVAs在淡水环境、污水处理厂及地表径流中被广泛检出，如Chen等^［6］^对中国太湖和钱塘江地表水样品中13种苯并三唑紫外稳定剂进行分析，其中UV-320、UV-326、UV-327、UV-328、2-（2′-羟基-5′-叔辛基苯基）苯并三唑（UV-329）和UV-350的检出平均质量浓度为8.77～16 ng/L，检出频率为14%～96%。OUVAs的普遍使用导致在水体^［7，8］^、沉积物^［9］^、灰尘^［10］^以及生物体^［11］^乃至人体^［12］^中均存在污染。多数OUVAs具有较强的亲脂性、低水溶性、光稳定性和抗生物降解性，可在多种环境介质中迁移，并通过食物链富集^［13］^。因此，其环境污染与生态风险已引起全球关注。毒性研究显示，OUVAs的急性毒性可能随其亲脂性增强而提高^［14］^，进一步加剧其对生物和人体健康的潜在风险。

准确分析饮用水中OUVAs的种类与含量，对评估其健康风险、解析生物效应及保障饮用水安全具有重要意义。目前，水中OUVAs的检测方法主要包括气相色谱-串联质谱法（GC-MS/MS）^［15］^和超高效液相色谱-串联质谱法（UHPLC-MS/MS）^［16］^等。现有文献中，基于UHPLC-MS/MS的OUVAs分析方法多聚焦于二苯甲酮类和苯并三唑类化合物，存在目标物覆盖有限、分析时间长、灵敏度不足及回收率不稳定等问题，难以实现多种OUVAs的高效同步检测。为此，本研究采用固相萃取（SPE）-UHPLC-MS/MS技术，通过系统优化前处理富集程序与色谱-质谱参数，建立了同时检测饮用水中16种OUVAs（包括9种紫外稳定剂与7种紫外过滤剂）的方法。该方法覆盖的目标物较广，分析快速，且灵敏度与准确度较高，为研究饮用水中OUVAs的分布特征和对人群的健康风险评估提供了可靠的技术支撑。

## 1 实验部分

### 1.1 仪器、材料与试剂

液相色谱-三重四极杆质谱系统：Ultra超高效液相色谱搭载HPMS-TQ三重四极杆质谱仪（华谱科仪北京科技有限公司），配备电喷雾离子源（ESI）；Fotector Plus型高通量全自动固相萃取仪（睿科仪器厦门有限公司）；NI-28型恒温水浴氮吹仪（上海屹尧公司）；PHSJ-4F型实验室pH计（上海仪电科仪股份有限公司）；VXMANAL型涡旋振荡器（常州奥豪斯仪器有限公司）；ME104/02型电子天平（感量0.000 1 g，瑞士Mettler公司）。0.22 μm聚四氟乙烯滤膜（美国PALL公司）；Oasis HLB固相萃取柱（200 mg/6 mL，美国Waters公司）。甲醇、乙酸铵、二氯甲烷、乙酸乙酯（LC-MS级，美国Fisher公司）；抗坏血酸（分析纯，美国Merck公司）；实验用水为超纯水（18.2 MΩ·cm）。

标准物质： 2-（2*H*-苯并三氮唑-2-基）对甲苯酚（UV-P）、2-羟基-4-甲氧基二苯甲酮（UV-9）、UV-320、UV-326、UV-327、UV-328、UV-329、UV-350、2-（2*H*-苯并三唑-2）-4，6-二（1-甲基-1-苯基乙基）苯酚（UV-234）、2-羟基-4-正辛氧基二苯甲酮（UV-531）、2-（2*H*-苯并三唑-2-基）-6-（1-甲基-1-苯乙基）-4-（1，1，3，3-四甲基丁基）苯酚（UV-928）、2-（4，6-二苯基-1，3，5-三嗪-2-基）-5-己基氧基-苯酚（UV-1577）、2，2′-羟基-4-甲氧基二苯甲酮（BP-8）、奥克立林（OC）、3-（4-甲基苯亚甲基）樟脑（4-MBC）、对二甲氨基苯甲酸异辛酯（ODPABA）、2-（2′-羟基-5′-叔辛基苯基）苯并三唑-^13^C_6_（UV-329-^13^C_6_）、2-羟基-4-甲氧基二苯甲酮-D_5_（UV-9-D_5_）、2-（2*H*-苯并三氮唑-2-基）对甲苯酚-D_3_（UV-P-D_3_）、2′-（2′-羟基-3′-叔丁基-5′-甲基苯基）-5-氯苯并三唑-D_3_（UV-326-D_3_）、2-（2′-羟基-3′，5′-二叔戊基苯基）苯并三唑-D_12_（UV-328-D_12_）。所有标准溶液均购自天津阿尔塔科技有限公司（质量浓度均为100 μg/mL）。

### 1.2 溶液配制

准确移取16种100 μg/mL OUVAs 单标准溶液各100 μL于1 mL容量瓶中，再以甲醇定容后混匀，配制质量浓度为1 μg/mL的混合标准溶液，于4 ℃避光保存。用甲醇将内标UV-329-^13^C_6_、UV-9-D_5_、UV-P-D_3_、UV-326-D_3_、UV-328-D_12_配制成质量浓度为100 μg/L的同位素内标混合储备液。实验开始前，取一定量的混合标准溶液，用甲醇将16种OUVAs的混合溶液稀释成质量浓度为5、10、20、25、50、100、200、250、500 μg/L的混合标准溶液，然后分别将1 mL各质量浓度的混合溶液与500 μL同位素内标混合储备液混合，以甲醇为溶剂稀释至10 mL，配制质量浓度为0.5、1、2、2.5、5、10、20、25、50、100 μg/L的OUVAs混合标准工作液，其中含有5 μg/L的同位素内标。所有溶液均于4 ℃避光保存。

### 1.3 样品采集和处理

使用1 L棕色玻璃瓶采集水样，采样前预先放水3~5 min以排除管道滞留水。水样采集时加入0.025 g抗坏血酸并混匀，于4 ℃条件下避光保存。准确量取500 mL水样，考虑到部分有机紫外吸收剂在碱性或中性条件下会发生水解反应而分解，而酸性条件可以有效地抑制这些水解反应，保持样品的完整性，加入盐酸调节pH至2.0~3.0^［17］^，再加入1 mL 5 μg/L的混合内标工作液。将HLB固相萃取柱依次经6 mL二氯甲烷、6 mL甲醇和12 mL超纯水进行活化与平衡；上样流速为8 mL/min，之后以12 mL超纯水淋洗。萃取柱经氮气干燥20 min后，采用12 mL二氯甲烷-乙酸乙酯（1∶1，体积比）混合溶液进行洗脱，收集洗脱液于15 mL聚丙烯离心管中。在30 ℃水浴中采用温和氮气流将洗脱液浓缩至近干，随后以甲醇复溶并定容至1 mL，涡旋混匀后使用0.22 μm孔径聚四氟乙烯滤膜对样品过滤后进行UHPLC-MS/MS分析。

### 1.4 仪器条件

UHPLC条件

色谱柱：Acquity Premier BEH C_18_色谱柱（100 mm×2.1 mm，1.7 μm，美国Waters公司）；柱温45 ℃；进样体积10 μL。流动相A为2 mmol/L乙酸铵水溶液，流动相B为甲醇，流速0.35 mL/min；梯度洗脱程序：0~4 min，流动相B由10%升至65%；4~6 min，流动相B升至95%，保持8 min；14.1 min时，流动相B快速降至10%并保持1.9 min。16 min内完成16种OUVAs的分离检测。

质谱条件

离子源为电喷雾电离（ESI），正离子扫描，以多反应监测（MRM）模式分析；离子化电压：5 500 V；离子源温度：400 ℃；反吹气：206.8 kPa；喷雾气：275.8 kPa；辅助加热气：310.3 kPa；碰撞气：551.6 kPa。其余质谱检测参数见表1。

**表1:** 16种OUVAs及5种内标的质谱参数

Compound	Abbr.	Retention time/min	Parent ion （*m/z*）	Daughter ion （*m/z*）	DP/V	CE/eV	Internal standard
2-（2*H*-Benzotriazol-2-yl）-4，6-bis（1-methyl-1-phenylethyl）-phenol	UV-234	10.42	448	119	60	45	UV-329-^13^C_6_
				370^∗^	60	33	
2-（2′-Hydroxy-3′，5′-di-*tert*- butylphenyl）benzotriazole	UV-320	10.28	324	212	35	35	UV-329-^13^C_6_
				268^∗^	35	31	
2-（5-Chlor-2*H*-benzotriazol-2-yl）-6-（1，1-dimethylethyl）-4-methyl phenol	UV-326	10.41	316	57	40	45	UV-326-D_3_
				260^∗^	40	27	
2-（2′-Hydroxy-3′，5′-di-*tert*-butylphenyl）-5-chlorobenzotrizole	UV-327	11.09	358	57	50	61	UV-328-D_12_
				302^∗^	50	34	
2-（2*H-*Benzotriazol-2-yl）-4，6-ditertpentyl-phenol	UV-328	10.95	352	71	55	44	UV-328-D_12_
				282^∗^	55	32	
2-（2*H*-Benzotriazol-2-yl）-4-（1，1，3，3-tetramethylbutyl）phenol	UV-329	9.40	324	57	45	53	UV-329-^13^C_6_
				212^∗^	45	38	
2-（2*H*-Benzotriazol-2-yl）-4-（*tert*-butyl）-6-（*sec*-butyl）phenol	UV-350	10.09	324	212	45	38	UV-329-^13^C_6_
				268^∗^	45	31	
2-Hydroxy-4-*n*-octoxybenzo-phenone	UV-531	9.14	327	137^∗^	40	42	UV-329-^13^C_6_
				105	40	48	
2-（2-Benzotriazolyl）-4-methylphenol	UV-P	7.20	226	107	25	27	UV-P-D_3_
				120^∗^	25	26	
2-（2*H*-Benzotriazol-2-yl）-6-（1-methyl-1-phenylethyl）-4-（1，1，3，3-tetramethylbutyl）pheno-l	UV-928	11.42	442	364^∗^	35	18	UV-P-D_3_
				252	35	28	
2-（2*H*-Benzotriazol-2-yl）-4-methyl-6-（2-propenyl）phenol	UV-9	6.29	229	105	30	26	UV-9-D_5_
				151^∗^	30	27	
2-（4，6-Diphenyl-1，3，5-triazin-2-yl）-5-［（hexyl）oxy］-phenol	UV-1577	12.60	426	104^∗^	20	66	UV-P-D_3_
				342	20	38	
2′-Dihydroxy-4-methoxybenzophenone	BP-8	5.71	245	121^∗^	40	22	UV-9-D_5_
				151	40	25	
2-Ethylhexyl 4-dimethylaminobenzoate	ODPABA	8.00	278	151^∗^	40	42	UV-329-^13^C_6_
				166	40	28	
3-（4-Methylbenzyliden）camphor	4-MBC	7.29	255	105^∗^	40	43	UV-9-D_5_
				97	40	26	
Octocrylene	OC	7.78	362	204	20	45	UV-329-^13^C_6_
				232^∗^	20	31	
				250	20	21	
UV-9-D_5_	*/*	6.25	234	151	30	28	*/*
UV-329-^13^C_6_	*/*	9.38	330	218	70	35	*/*
UV-P-D_3_	*/*	7.18	229	110	40	28	*/*
UV-326-D_3_	*/*	10.36	319	263	40	28	*/*
UV-328-D_12_	*/*	10.95	364	288	50	35	*/*

DP： declustering potential； CE： collision energy； ∗ quantitative ion； /： no value.

### 1.5 质量控制

每次实验开始前及结束后，均对实验环境进行彻底清洁，以避免在后续实验中引入目标分析物，确保实验的准确性和可重复性。鉴于OUVAs在日常生活和生产中广泛应用，且常直接接触人体皮肤，为最大程度降低过程污染，我们采取了以下措施：整个实验过程中操作人员均佩戴丁腈手套；实验人员尽量避免使用含有目标OUVAs的个人护理产品、衣物及配饰等。此外，在样品前处理与定量分析阶段，每批样品均设置程序空白，以监测实验过程中可能存在的污染情况。

## 2 结果与讨论

### 2.1 色谱条件优化

液相色谱系统中常用的有机试剂为甲醇、乙腈，因此比较了甲醇-水、乙腈-水、甲醇-水（含0.1%甲酸）、甲醇-水（含2 mmol/L乙酸铵）4种流动相在相同条件下对各目标物色谱峰响应的影响。结果表明，添加乙酸铵的甲醇-水体系能显著改善峰形与响应稳定性，故最终选用该体系作为流动相。16种OUVAs的色谱图见图1，各化合物分离良好，保留时间见表1。

**图1:**
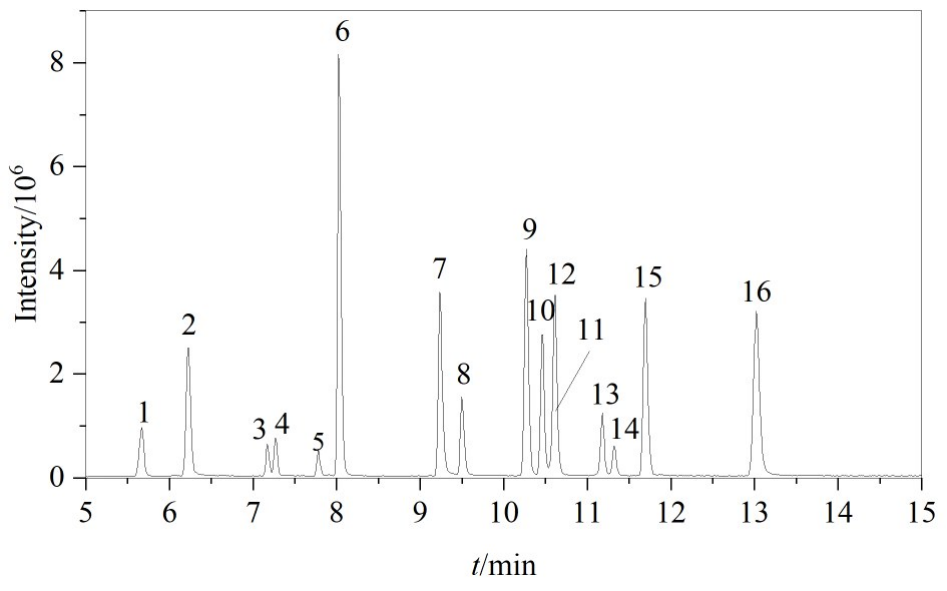
16种OUVAs混合标准溶液的色谱图（20 μg/L）

### 2.2 前处理条件的优化

#### 2.2.1 固相萃取柱的选择

在500 mL水样中加入混合标准溶液至终质量浓度为20 ng/L，根据目标化合物性质和固相萃取柱填料成分，考察了HLB柱（200 mg/6 mL，美国Waters公司）、Plexa柱（200 mg/6 mL，美国Agilent公司）、C_18_柱（200 mg/6 mL，美国Waters公司）对水样中OUVAs的萃取效果，计算各目标化合物的回收率。结果如图2所示，HLB柱对目标化合物的萃取效果最好，回收率最高，因此，选择HLB固相萃取小柱对水样进行萃取富集。

**图2:**
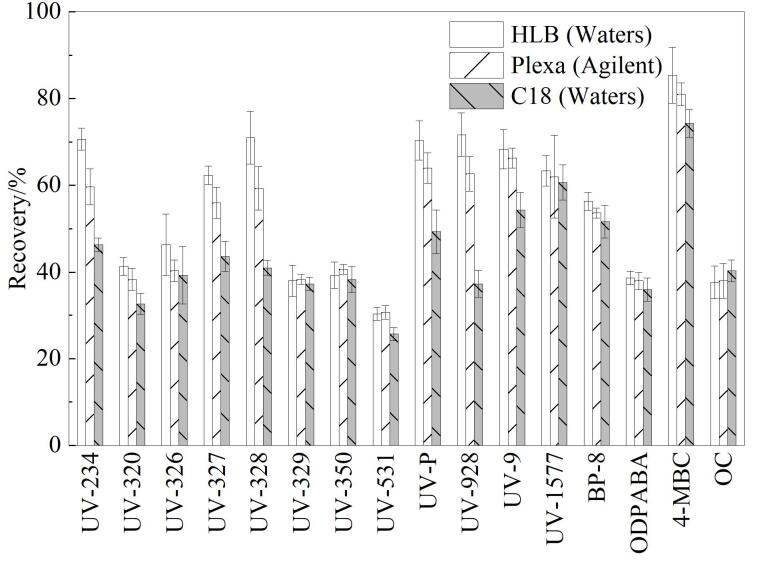
固相萃取柱类型对16种OUVAs回收率的影响（*n*=6）

#### 2.2.2 加入抗坏血酸浓度的选择

抗坏血酸具有螯合金属离子的作用，可减少金属络合物干扰，同时作为强还原剂和抗氧化剂，抗坏血酸能有效清除溶解氧和自由基，保护易氧化的目标分析物不被降解，从而维持其原始浓度和质谱响应。在前处理过程中比较了4种质量浓度的抗坏血酸（0、50、100、200 mg/L）对目标化合物回收率的影响。结果发现，在添加50 mg/L的抗坏血酸时，目标化合物的回收率最高，当抗坏血酸浓度更高时，回收率没有明显变化，结果如图3所示。因此，最终选择加入50 mg/L抗坏血酸。

**图3:**
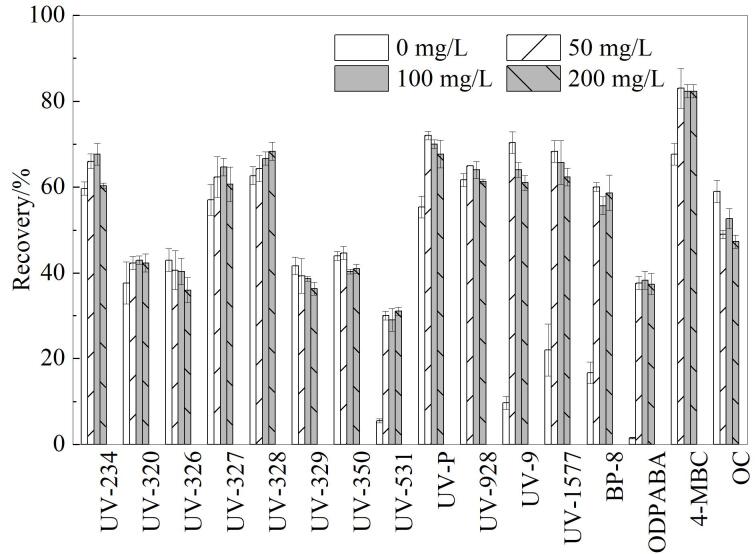
抗坏血酸质量浓度对16种OUVAs回收率的影响（*n*=6）

#### 2.2.3 上样流速的选择

选用500 mL水样、12 mL二氯甲烷-乙酸乙酯（1∶1，体积比）进行洗脱的条件下，考察了3种上样流速（6、8和10 mL/min），结果如图4所示。大部分目标物质在8 mL/min上样流速下回收率较好，原因可能是上样流速过高时，固相萃取柱无法有效吸附目标化合物，导致目标化合物的保留不足。因此，最终选择8 mL/min流速作为最佳进样流速。

**图4:**
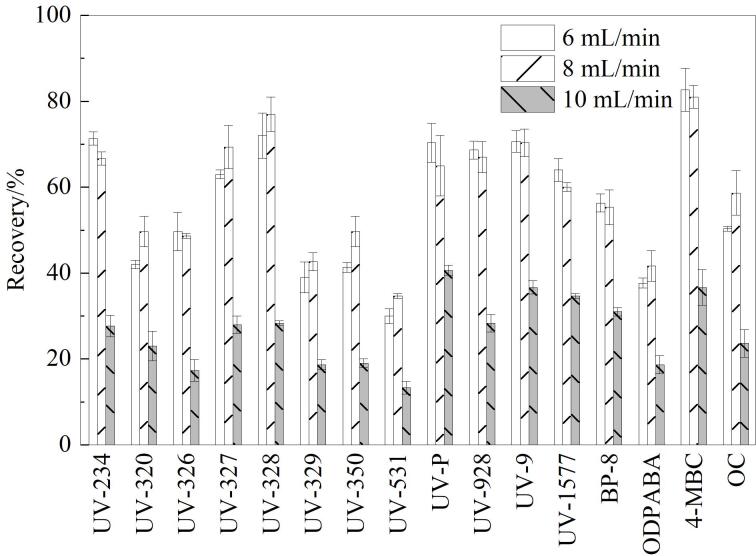
不同流速对16种OUVAs回收率的影响（*n*=6）

#### 2.2.4 洗脱剂体积的选择

进一步考察了洗脱剂体积（8、12、16 mL）对水中各OUVAs回收率的影响。由图5可知，当洗脱剂体积逐渐增加时，OUVAs回收率表现出先上升后平稳的趋势。当洗脱剂体积为8 mL时，对目标化合物的洗脱效果不完全；当洗脱剂体积在12 mL时，洗脱效果较好，回收率达到最大；当洗脱剂体积超过12 mL时，回收率趋于平稳。因此，选择洗脱剂体积为12 mL。

**图5:**
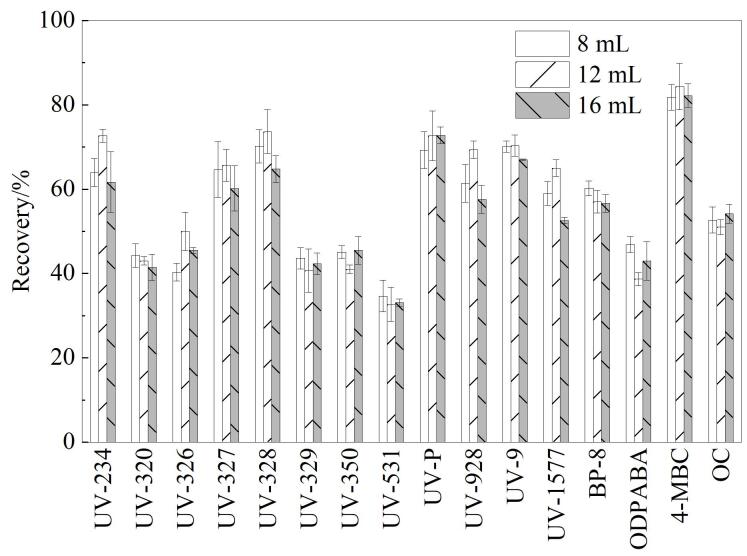
洗脱剂体积对16种OUVAs回收率的影响（*n*=6）

#### 2.2.5 氮吹温度的选择

氮吹温度可直接影响目标化合物的回收率。为减少氮吹步骤引起的目标化合物损失，实验对比了16种OUVAs在不同氮吹温度（30、35、40 ℃）下的回收率。结果表明，在30 ℃下氮吹时，目标化合物的回收率最高，结果如图6所示。因此，最佳氮吹温度为30 ℃。

**图6:**
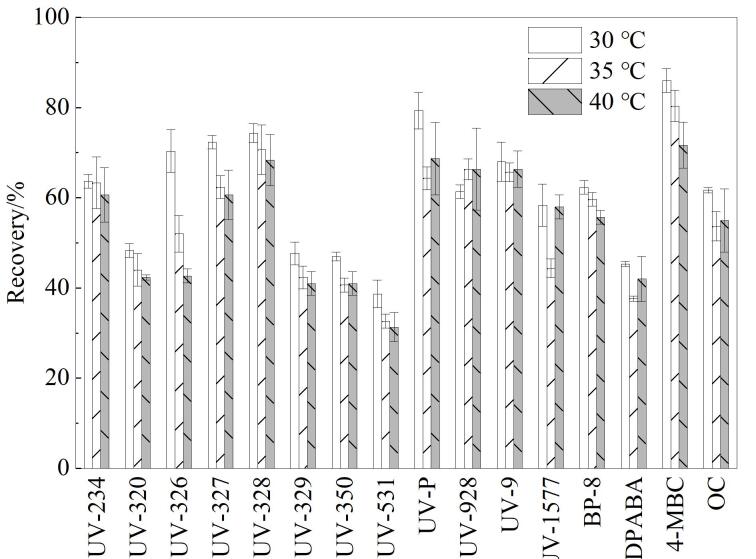
氮吹温度对16种OUVAs回收率的影响（*n*=6）

### 2.3 方法学考察

#### 2.3.1 线性范围、检出限和定量限

将系列混合标准工作液按优化后的UHPLC-MS/MS条件进行测定，对16种OUVAs的线性关系进行考察。为确定方法检出限（MDL）与方法定量限（MQL），根据HJ 168-2020方法检出限测定要求，向空白饮用水基质中添加低浓度目标物，制备7个平行加标样品，经完整前处理后分别上机测定，计算7次测定结果的标准偏差（SD），以3倍SD对应的浓度作为方法检出限（MDL），以10倍SD对应的浓度作为方法定量限（MQL），结果见表2。实验结果表明，16种OUVAs在相应线性范围内均表现出良好的线性关系，相关系数均大于0.992。该方法的检出限与定量限较低，适用于饮用水中16种OUVAs的分析检测。

**表2:** 16种OUVAs的线性范围、线性方程、相关系数、方法检出限和方法定量限

Compound	Linear range/（μg/L）	Linear equation	*r*	MDL/（ng/L）	MQL/（ng/L）
UV-234	0.5-100	*Y*=1.20686*X*+0.17122	0.996	0.03	0.1
UV-320	0.5-100	*Y=*0.44997*X*-0.09580	0.997	0.15	0.5
UV-326	10-100	*Y=*0.15718*X+*0.23251	0.997	2.5	7.5
UV-327	1-100	*Y*=0.81915*X*-0.06586	0.998	1	2.5
UV-328	0.5-100	*Y*=2.53184*X*+0.01168	0.999	0.15	0.5
UV-329	0.5-100	*Y*=0.16739*X*-0.02292	0.996	0.1	0.25
UV-350	0.5-100	*Y*=0.57453*X*-0.06217	0.998	0.1	0.3
UV-531	0.5-100	*Y*=1.25123*X+*0.22148	0.998	0.1	0.3
UV-P	15-100	*Y*=0.23956*X*+0.11548	0.996	5	15
UV-928	0.5-100	*Y*=2.70003*X*+0.47397	0.995	0.03	0.1
UV-9	0.5-100	*Y*=0.25082*X*+0.02903	0.998	0.1	0.3
UV-1577	0.5-100	*Y*=2.25958*X*+1.42323	0.993	0.03	0.1
BP-8	0.5-100	*Y*=0.12690*X*+0.00524	0.999	0.15	0.5
ODPABA	0.5-100	*Y*=1.39631*X*+0.45531	0.992	0.1	0.3
4-MBC	2-100	*Y*=0.05848*X*+0.0003969	0.998	1.5	5
OC	2-100	*Y*=0.07564*X*-0.01247	0.996	1.5	5

*Y*： peak area ratio of target compound to the corresponding internal standard； *X*： mass concentration， μg/L.

#### 2.3.2 方法的准确度和精密度

采用所建立的方法，以饮用水为基质进行了加标回收试验以评估准确度与精密度。在空白水样中分别进行3个水平的加标试验，每个浓度水平在1 d内平行测定6次，并使用内标法进行校准，将处理后目标化合物与相应内标的定量离子峰面积的比值带入标准曲线，计算实际测得浓度，除以富集倍数后，与添加到样品中的加标浓度进行比较，得到绝对回收率并计算精密度；在空白水样中再分别进行3个水平的加标试验，每个浓度水平在6 d内平行测定，计算绝对回收率和日间精密度。结果（表3）表明，16种OUVAs在不同加标水平下的平均回收率为75.0%~130.6%，所有化合物的回收率均高于70%；相对标准偏差（RSD）为0.9%~12.9%，该方法具有良好的准确度和精密度。

**表3:** 3个加标水平下16种OUVAs的回收率和精密度（*n*=6）

Compound	5 ng/L			20 ng/L			50 ng/L		
	Recovery/%	Inter-day RSD%	Intra-day RSD%	Recovery/%	Inter-day RSD%	Intra-day RSD%	Recovery/%	Inter-day RSD%	Intra-day RSD%
UV-234	125.5	3.1	6.5	130.6	3.7	2.3	119.4	3.5	2.2
UV-320	96.9	8.4	6.9	91.0	2.9	7.3	96.6	2.3	4.6
UV-326	80.9	8.9	6.5	84.4	3.1	2.8	91.6	3.6	4.5
UV-327	85.6	6.3	6.5	85.6	5.3	5.5	85.3	7.8	10.5
UV-328	91.6	5.9	4.9	91.3	3.7	7.3	91.6	4.2	7.2
UV-329	107.2	4.1	5.7	91.4	1.1	6.7	80.4	1.6	7.7
UV-350	104.6	7.3	7.9	92.9	5.1	3.2	76.2	4.6	5.6
UV-531	76.7	12.9	10.5	90.1	6.8	3.8	86.5	3.3	8.7
UV-P	110.1	5.9	8.6	105.9	3.7	2.7	99.6	4.9	5.1
UV-928	92.2	5.7	3.7	98.2	6.4	9.1	97.1	3.9	5.9
UV-9	119.6	4.8	5.8	109.9	0.9	5.2	110.8	2.9	6.6
UV-1577	106.3	9.8	7.6	114.9	6.0	5.1	113.4	6.1	5.4
BP-8	109.9	6.6	7.0	96.9	2.5	3.3	104.7	9.8	9.9
ODPABA	121.3	6.1	5.2	113.9	7.5	6.3	119.7	6.3	6.6
4-MBC	115.9	2.6	5.3	112.3	2.6	3.8	117.5	3.8	3.9
OC	82.1	6.2	7.6	83.7	3.7	5.9	75.0	4.4	8.4

### 2.4 方法对比

将本研究所建立的方法与文献中其他饮用水中OUVAs的检测方法进行比较（表4）。结果表明，本方法在保证较低定量限以满足检测需求的同时，实现了对饮用水中16种OUVAs的高通量同步检测，解决了UV-326、UV-327等有机紫外稳定剂响应值较低的问题，且单次仪器分析时长仅需16 min，显著提升了检测效率。

**表4:** 本方法与其他方法的比较

Numbers of target compounds			Detection methods	Water source types	MQL/（ng/L）	Ref.
Organic UV filters	Organic UV stabilizers	Total				
5	11	16	SPE-UHPLC-MS/MS	drinking water	0.1-15	this study
5	0	5	SPE-GC-EI-MS	sea water， wastewater	0.02-8.42	［^18^］
8	0	8	Online SPE-LC-MS/MS	sea water	0.5-11.7	［^19^］
7	1	8	SPE-UHPLC-MS/MS	sea water	0.02-25.2	［^20^］
0	7	7	SBSE UHPLC-MS/MS	surface water	61.5-184	［^21^］

SBSE： stir bar sorption extraction.

### 2.5 实际水样的测定

采用建立的方法对7份水源水和7份饮用水进行检测，结果表明，UV-9、OC共2种有机紫外吸收剂被检出。在7份水源水样品中，有5份水样检出UV-9，7份水样检出OC，UV-9的检出质量浓度为<MQL~13.4 ng/L，检出率为71.4%，OC的检出质量浓度为5.0~32.23 ng/L；7份饮用水样品中，有1份水样检出UV-9，3份水样检出OC，UV-9的检出质量浓度为0.4 ng/L，检出率为14.3%，OC的检出质量浓度为<MQL~13.2 ng/L，检出率为42.8%。实验结果说明，水源水和饮用水中的UV-9和OC检出较为普遍，检出含量也较高。

## 3 结论

本研究建立了SPE-UHPLC-MS/MS同时检测饮用水中16种OUVAs的分析方法，通过优化前处理条件和质谱条件，使得目标化合物得到了较好的分离效果。该方法具备简便快速、环保、重复性好等优点，同时结果也表明该方法的回收率和精密度良好，可用于我国饮用水中多种OUVAs的监测。实际样品检测结果表明，水源水中普遍存在OUVAs污染，饮用水中OUVAs污染较少，可为后续评估饮用水中OUVAs的潜在危害提供可靠的技术支持。
